# An integrative analysis uncovers a new, pseudo-cryptic species of Amazonian marmoset (Primates: Callitrichidae: *Mico*) from the arc of deforestation

**DOI:** 10.1038/s41598-021-93943-w

**Published:** 2021-08-02

**Authors:** Rodrigo Costa-Araújo, José S. Silva-Jr., Jean P. Boubli, Rogério V. Rossi, Gustavo R. Canale, Fabiano R. Melo, Fabrício Bertuol, Felipe E. Silva, Diego A. Silva, Stephen D. Nash, Iracilda Sampaio, Izeni P. Farias, Tomas Hrbek

**Affiliations:** 1 Museu Paraense Emílio Goeldi, Mastozoology Collection, Belém, 66077-830 Brazil; 2 Laboratory of Evolution and Animal Genetics, Federal University of Amazonas, Manaus, 69077-000 Brazil; 3 School of Science, Engineering and Environment, University of Salford, Salford, M54WT UK; 4 Institute of Biosciences, Federal University of Mato Grosso, Cuiabá, 78060-900 Brazil; 5 Institute of Natural, Human and Social Sciences, Federal University of Mato Grosso, Sinop, 78557-267 Brazil; 6 Department of Forest Engineering, Federal University of Viçosa, Viçosa, 36570-900 Brazil; 7 Research Group on Primate Biology and Conservation, Mamirauá Institute for Sustainable Development, Tefé, 69553-225 Brazil; 8 Graduate Program in Ecology and Conservation, State University of Mato Grosso, Nova Xavantina, 78690-000 Brazil; 9 Departments of Anatomical Sciences and Art, Stony Brook University, Stony Brook, NY 11794 USA; 10 Institute of Coastal Studies, Federal University of Pará, Bragança, 68600-000 Brazil; 11 Department of Biology, Trinity University, San Antonio, 78212 USA

**Keywords:** Zoology, Phylogenetics, Taxonomy, Phylogenomics, Evolutionary biology

## Abstract

Amazonia has the richest primate fauna in the world. Nonetheless, the diversity and distribution of Amazonian primates remain little known and the scarcity of baseline data challenges their conservation. These challenges are especially acute in the Amazonian arc of deforestation, the 2500 km long southern edge of the Amazonian biome that is rapidly being deforested and converted to agricultural and pastoral landscapes. Amazonian marmosets of the genus *Mico* are little known endemics of this region and therefore a priority for research and conservation efforts. However, even nascent conservation efforts are hampered by taxonomic uncertainties in this group, such as the existence of a potentially new species from the Juruena–Teles Pires interfluve hidden within the *M. emiliae* epithet. Here we test if these marmosets belong to a distinct species using new morphological, phylogenomic, and geographic distribution data analysed within an integrative taxonomic framework. We discovered a new, pseudo-cryptic *Mico* species hidden within the epithet *M. emiliae*, here described and named after Horacio Schneider, the pioneer of molecular phylogenetics of Neotropical primates. We also clarify the distribution, evolutionary and morphological relationships of four other *Mico* species, bridging Linnean, Wallacean, and Darwinian shortfalls in the conservation of primates in the Amazonian arc of deforestation.

## Introduction

There are 146 primate species and subspecies in Amazonia, representing 20% of the global primate diversity^1^ and comprising the most diverse primate fauna in the world^2^. Nonetheless, primate diversity remains understudied in Amazonia, as manifested by regular discoveries of new species^3–5^. This incomplete taxonomic knowledge and the scarcity of basic ecological and distributional data for even well-known species is a major impediment to the design and implementation of effective conservation actions^6^. Bridging these substantial Linnean, Wallacean, and Darwinian shortfalls is also a pre-requisite to understanding the biotic and abiotic drivers of the evolutionary history of Neotropical primates^7^.

The Amazonian arc of deforestation concentrates nearly one-third of all global deforestation^8,9^. It also harbours 52 primate species—over one third of Amazonian primates—of which 42% are threatened with extinction according to the IUCN^10^ (Supplementary Table S1). Therefore, research and conservation efforts on the primates of the arc of deforestation are priority within the Neotropics.

Endemic to this region are marmosets of the genus *Mico*^3,11^. One of the main taxonomic, distributional, and evolutionary uncertainties—the Linnean, Wallacean and Darwinian shortfalls, respectively—in *Mico* species concern the Snethlage’s marmoset *M. emiliae*^12^. The pelage colour of this species has confounded researchers for a century hindering accurate assessments of the taxonomy and distribution of this and other five *Mico* species, as well as the assessment of species diversity in this genus.

**Figure 1:**
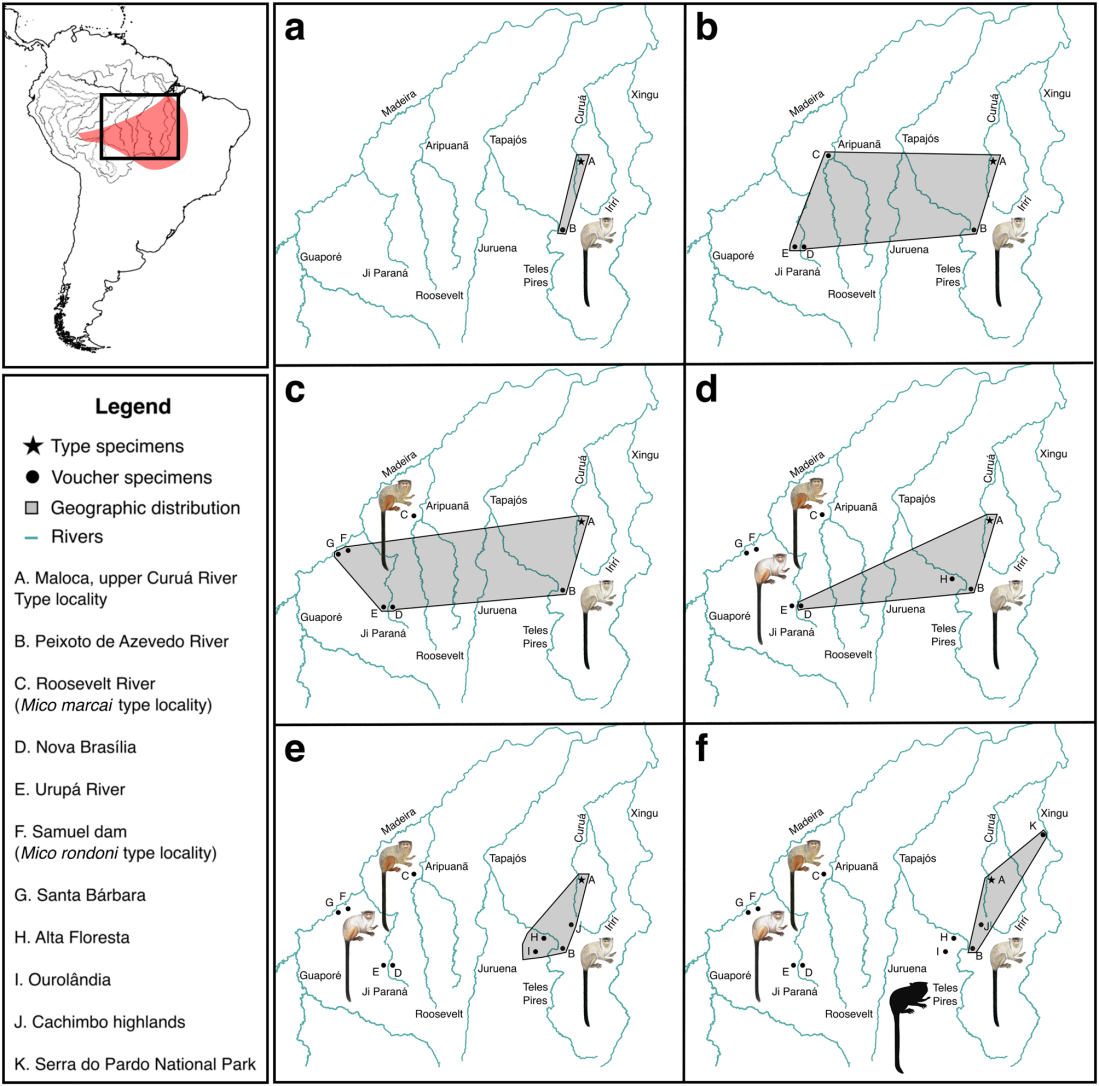
History of hypotheses on the geographical distribution of *Mico emiliae* according to past taxonomic and phylogenetic studies. The outer map of South America shows the boundaries of Amazonia biome with main riverine configuration in black and the arc of deforestation in red. The grey area in the inner maps shows the geographical distribution of *M. emiliae*: **(a)** 1920–1990: *M. emiliae* as either valid species, invalid species, or subspecies of *M. argentatus*^12–18^; **(b)** 1991–1992: a valid species occurring on Tapajós–Xingu, Guaporé–Ji-Paraná and Ji-Paraná–Aripuanã interfluves^19^; **(c)** 1993–2001: description of *M. marcai* for part of the Ji-Paraná–Aripuanã interfluve, and *M. emiliae* as a subspecies of *M. argentatus* with occurrence on the three interfluves of b^20^; **(d)** 2002–2010: description of *M. rondoni* for part of the Guaporé–Ji-Paraná interfluve and *M. emiliae* as a valid species occurring on the Juruena–Teles Pires, Ji-Paraná–Aripuanã and Tapajós–Xingu interfluves^21^; **(e)** current hypothesis supported by taxonomic studies^21,22^; **(f)** hypothesis proposed since 1993 by A. Rylands and colleagues^11,23–25^ (see Supplementary Table S2 for localities coordinates). Illustrations: Stephen Nash.

The description of *M. emiliae* in 1920 was based on the pelage colour of two specimens collected at “Maloca, upper Curuá River”^12^—an imprecise locality in the Tapajós–Xingu interfluve, southern Amazonia, Brazil. Researchers debated for 70 years if *M. emiliae* was distinct from *M. argentatus*^13–18^ until both were accepted as separate species based on patterns of pelage colouration^19^. Nonetheless, additional taxonomic confusion has arisen concerning the identification of marmosets from three interfluves—Guaporé–Ji-Paraná, Ji-Paraná–Aripuanã, and Juruena–Teles Pires (Fig. 1)—as *M. emiliae* based on an apparent similarity in pelage colour patterns of these marmosets.

The marmosets from the Ji-Paraná–Aripuanã and from the Guaporé–Ji-Paraná interfluves are currently considered two distinct species, *M. marcai*^20^ and *M. rondoni*^21^, respectively. However, the eight specimens collected in 1995 from two localities in the Juruena–Teles Pires interfluve and stored in the Museu Paraense Emílio Goeldi (Goeldi’s Museum) have been classified as *M. emiliae* by some authors^21,22^, whereas others have considered only the Tapajós–Xingu marmosets as *M. emiliae*^11,23–25^, casting into doubt the taxonomic identity of the Juruena–Teles Pires marmosets.

To date, *M. emiliae*, *M. marcai*, *M. rondoni*, and the Juruena–Teles Pires were not subjected to morphological or molecular studies with enough specimens to permit the differentiation of population from species-level diversity. Primate species are known to be limited by rivers in Amazonia^26^ and thus it is highly likely that the Juruena–Teles Pires marmosets represent a distinctive species—and, if that were the case, this taxon would occur in an area of intense deforestation and thus likely to be threatened with extinction^4,27^.

As part of the findings from an ongoing research on ecology, evolution, and systematics of Amazonian marmosets, which has as one of the main goals to clarify the taxonomy of the Juruena–Teles Pires marmosets, we found that these marmosets present a cohesive pelage colour pattern that is also distinctive from *M. emiliae* and other geographical neighbour taxa. Here we test whether the Juruena–Teles Pires marmosets represent a species distinct from *M. emiliae*, as well as *M. argentatus*, *M. marcai*, and *M. rondoni*. We conducted field expeditions to collect new distribution records, tissue samples and specimens, and examined specimens in museums, including all type and voucher specimens of *M. emiliae*, *M. marcai*, *M. rondoni*, and Juruena–Teles Pires marmosets plus numerous *M. argentatus* specimens. We then generated new morphological, phylogenomic and distribution data, which were analysed and interpreted within an integrative taxonomic framework.

## Methods

### Integrative approach in taxonomic hypothesis-testing and decision-making

We adopted an integrative approach^28^ to test the hypothesis that Juruena–Teles Pires marmosets belong to a species distinctive from *M. emiliae*, and to test whether *M. marcai*, *M. rondoni*, and *M. argentatus* are valid species. Integrative taxonomy provides an objective framework to test species hypotheses and accommodates practical constraints of data collection and analyses, resulting in accurate taxonomic decisions and more stable classifications––especially when morphology and nuclear DNA data are used^28,29^. Here we use pelage colour, nuclear genomic DNA, and distribution datasets, each associated with specific criteria to refute or to accept the null hypothesis (Supplementary Table S3), following the monophyly and diagnosability conceptualization of the phylogenetic species concept^30^.

### Fieldwork

Between 2015 and 2018, we conducted ten field expeditions across southern Amazonia to obtain new distribution records, specimens, and samples to overcome the previous scarcity of these materials and data in museums and in literature. The surveys consisted of trekking in the forest or canoeing up streams, while playing long-calls of *M. marcai* to stimulate vocalization and approximation of marmosets and increase detection probabilities^3^. For each observation we registered the exact location with a GPS device and the type of habitat. The collection of specimens and samples of muscular tissue were carried out with a permit from the Chico Mendes Institute of Biodiversity Conservation (permit number 50416), the federal institution that regulates biodiversity research in Brazil. Specimen sampling followed the protocols established for studying primates in protected areas of Amazonia^31^ and the code of best practices for field primatology of the International Primatological Society (http://www.internationalprimatologicalsociety.org/policy.cfm). Specimens were stored in the mammal collections of the National Institute of Amazonian Research and Goeldi’s Museum; tissue samples were preserved in 96% ethanol in the Animal Genetics Tissue Bank of the Federal University of Amazonas. No specimen was subjected to experimental conditions or protocols.

### Morphology

We collected data of pelage colour of 10 chromogenetic fields^32^ (Supplementary Fig. S4) through the direct examination of 598 skins of specimens obtained in the field and stored in museums encompassing all known species of the genera *Callibella* and *Mico* (Supplementary Table S5). The chromogenetic fields are informative morphological characters because they summarize most of the variation in the pelage colouration and provide an objective basis for comparisons in marmosets^3,18^ and other primates^4,18,33^. We also examined tegument colour on the face and ears, and hairiness of the ears to delimit supra-specific lineages as these characters are synapomorphies of four species groups of *Mico*^3^. Based on these four species groups of *Mico* and morphological synapomorphies, we expected (i) marmosets from Juruena–Teles Pires interfluve to form a lineage together with *M. marcai* and *M. melanurus*; (ii) *M. argentatus*, *M. emiliae*, *M. intermedius*, *M. leucippe*, *M. munduruku*, and *M. rondoni* to form a second lineage; (iii) *M. humeralifer* and *M. mauesi* to form a third lineage; and (iv) *M. saterei* to form a fourth, single-species lineage.

### Phylogenomics

After DNA extraction from muscular tissue samples^34^, we used a modified protocol of ddRAD sequencing^35^ optimized for the IonTorrent PGM that permits simultaneous digestion, ligation, and barcoded adapter incorporation (https://github.com/legalLab/protocols-scripts)^36^. Samples were sequenced on the IonTorrent PGM using the manufacturer’s recommended protocol and the sequencing reads were processed using the pyRAD pipeline^37^. For the de novo assembly, we used a minimum coverage of 5 × per locus, assembling all fragments of 320–400 base pairs. Nucleotides with PHRED scores < 30 were excluded, as well as loci with more than three low-quality nucleotides. Following demultiplexing and extraction of loci using the above criteria, we proceeded with clustering of alleles within loci and of loci across individuals. We generated a dataset for downstream analyses that included all individuals and loci present in at least 50% of the samples^38^, which was subjected to PartitionFinder2^39^ to estimate the optimal number of partitions. For Bayesian Inference analysis carried out in BEAST2^40^, site models, clock models and trees of each partition were unlinked. Site models were implemented based on the PartitionFinder2 results, and all partitions were allowed to evolve under an uncorrelated lognormal model with clock rates and standard deviations of clock rates being estimated. We ran the MCMC for 10^9^ generations, collecting 5 × 10^4^ samples. We carried out partitioned Maximum Likelihood analysis in RAxML^41^ with site models for each partition based on the PartitionFinder2 results. We sampled the genomes of representative species from the four *Mico* lineages as delimited in the morphology section, to have a representation of all four major lineages of *Mico* in the analyses. We sampled multiple individuals of the Juruena–Teles Pires marmosets and of the other species of the same lineage, which included *M. marcai*, as well as the other species previously confounded with *M. emiliae* (*M. argentatus* and *M. rondoni*); we used *Callibella humilis*, *Cebuella niveiventris*, and *Callithrix jacchus* as outgroups (Supplementary Table S6). We consider as strong support for Bayesian inference a posterior probability ≥ 0.95 and the equivalent bootstrap proportion for Maximum Likelihood inference (≥ 70%)^42^.

We then carried out a path sampling analysis in BEAST2 focusing on *M. argentatus*, *M. emiliae*, and *M. leucippe*, whose phylogenetic relationships are unresolved according to our results and to a previous study^3^. The objective was to investigate if the observed lack of monophyly is compatible with incomplete lineage sorting, hybridization or the existence of polymorphic species, i.e. a species that encompass others currently recognized as distinctive based on pelage colour patterns. Using DiscoSnp-RAD^43^, we extracted single nucleotide polymorphisms (SNPs) from our reads using a minimum read depth of 5. The highest quality SNPs were then sampled from each locus and the SNPs were further filtered on quality. We retained only those SNPs with rank > 0.9––a statistic incorporating the discriminant power and read coverage of each SNP––and those that were present in at least 90% of the samples. We then used path sampling in BEAST2, and collected marginal probabilities of alternate taxonomic hypotheses: one species (*M. emiliae-leucippe-argentatus*), two species (*M. argentatus* and *M. emiliae-leucippe*; *M. emiliae* and *M. leucippe-argentatus*; *M. leucippe* and *M. emiliae-argentatus*) and the current hypothesis of three species (*M. emiliae*, *M. leucippe*, and *M. argentatus*). Marginal probabilities of the competing taxonomic hypotheses were then compared by Bayes factors^44^.

### Distribution

All the localities of *Mico* specimens collected in the field and examined in museums were georeferenced and grouped according to their morphotypes on a relief map (vegetation type, altitude, river basins) to explore potential geographical barriers and areas of gene flow using QGIS^45^.

## Results

### Morphology

**Figure 2:**
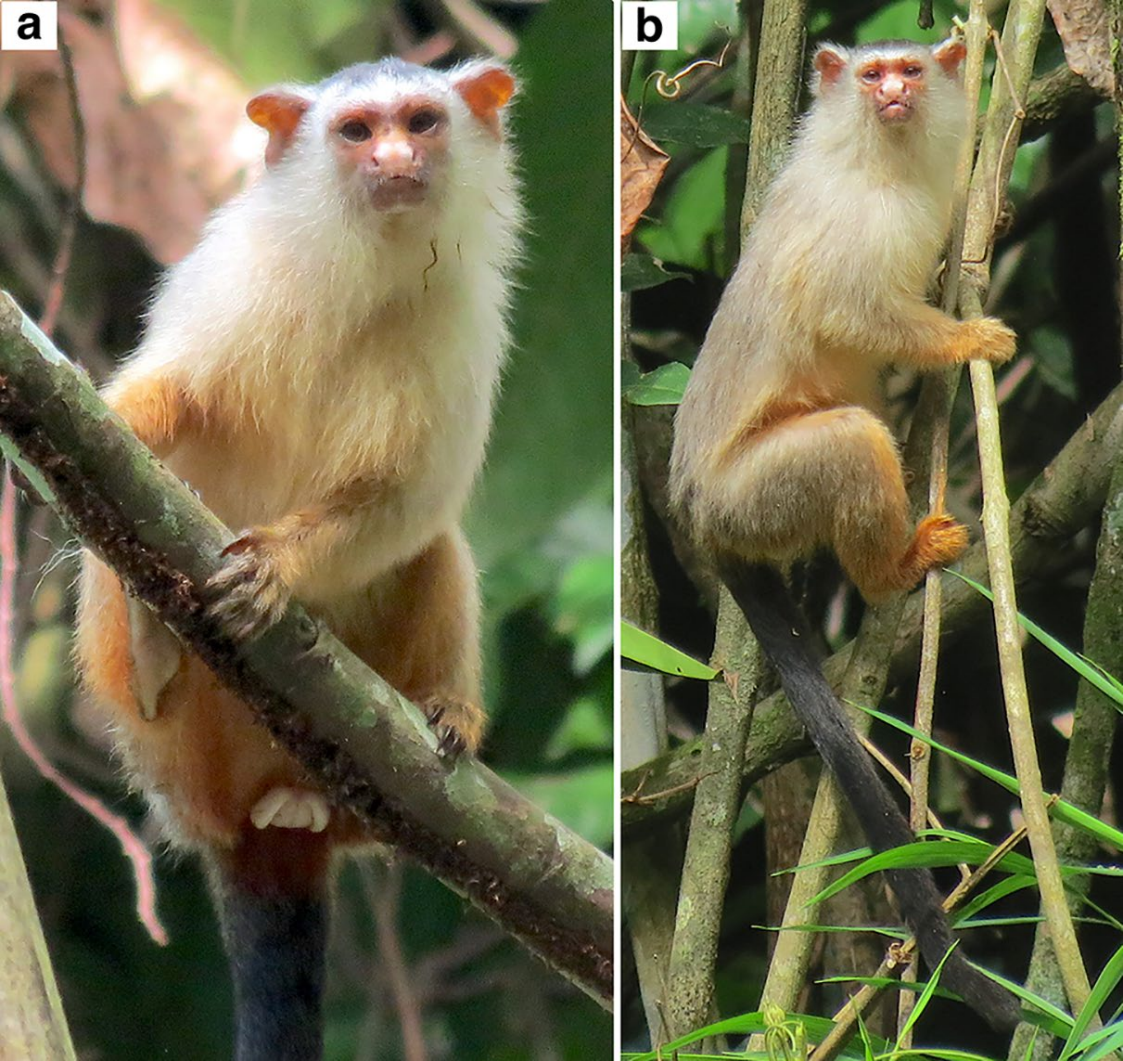
Schneider’s marmosets *Mico schneideri* sp. n. recorded at the type locality: Paranaíta, left margin of the Teles Pires River, Mato Grosso State, Brazil. **(a)** Adult female; **(b)** adult male. Photos: Diego Silva.

**Figure 3:**
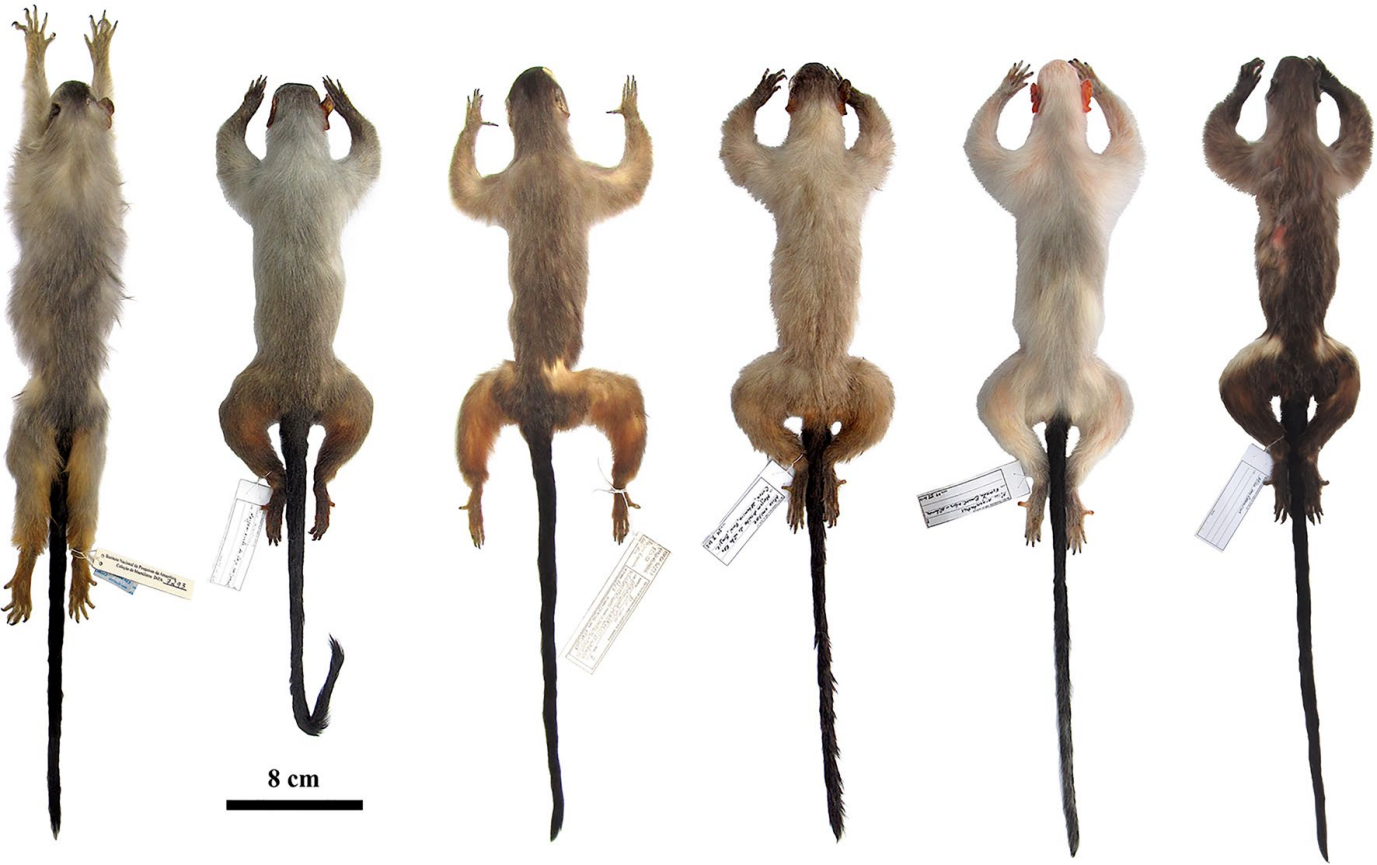
Dorsal view of skins of *Mico* species tested for diagnosability of morphological characters of pelage colour. Left to right: *Mico schneideri* sp. n. holotype (INPA 7293), *M. rondoni* (MPEG 45620), *M. marcai* (MPEG 42807), *M. emiliae* (MPEG 45566), *M. argentatus* (MPEG 45609), and *M. melanurus* (MPEG 45571).

All the marmosets from the Juruena–Teles Pires interfluve have discrete and objectively identifiable diagnostic states in pelage colour characters: the uniform lead coloration of saddle and rump and the cream-silvery underparts, which present orangish hues in living specimens (Fig. 2), are autapomorphies that readily distinguish these marmosets from *M. emiliae* and all other *Mico* species. The Juruena–Teles Pires marmosets, *M. argentatus*, *M. emiliae*, *M. marcai*, *M. melanurus*, and *M. rondoni* are clearly diagnosable in terms of pelage colour (Table 1; Fig. 3).

### Phylogenomics

**Figure 4:**
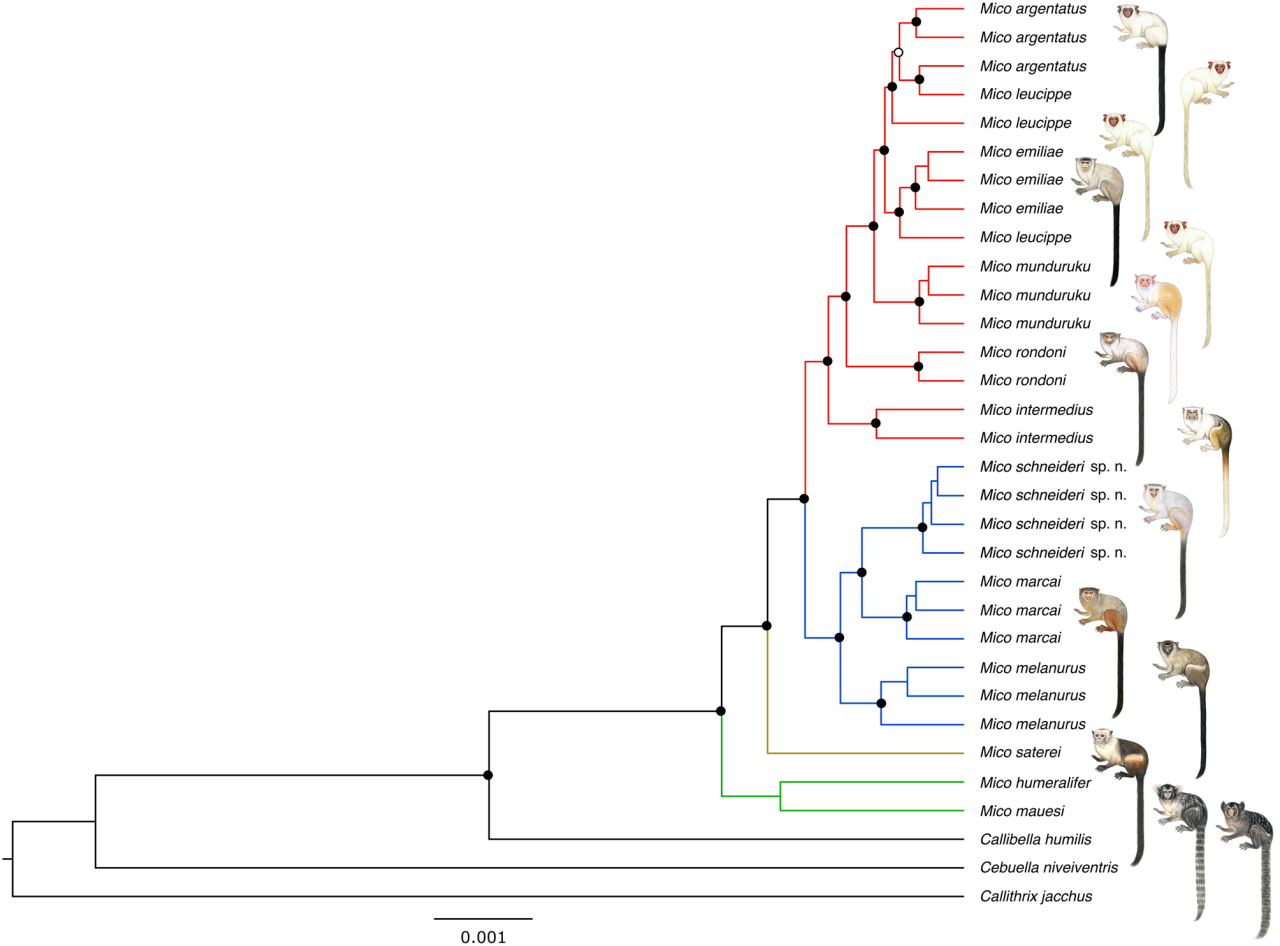
Bayesian phylogeny of the genus *Mico* inferred with ddRAD data, indicating the four main lineages of this genus in distinct colours (black lines are outgroups). Clades supported are indicated by black circles and unresolved branches (≤ 0.95 posterior probability) by white circles. Illustrations: Stephen Nash.

**Figure 5:**
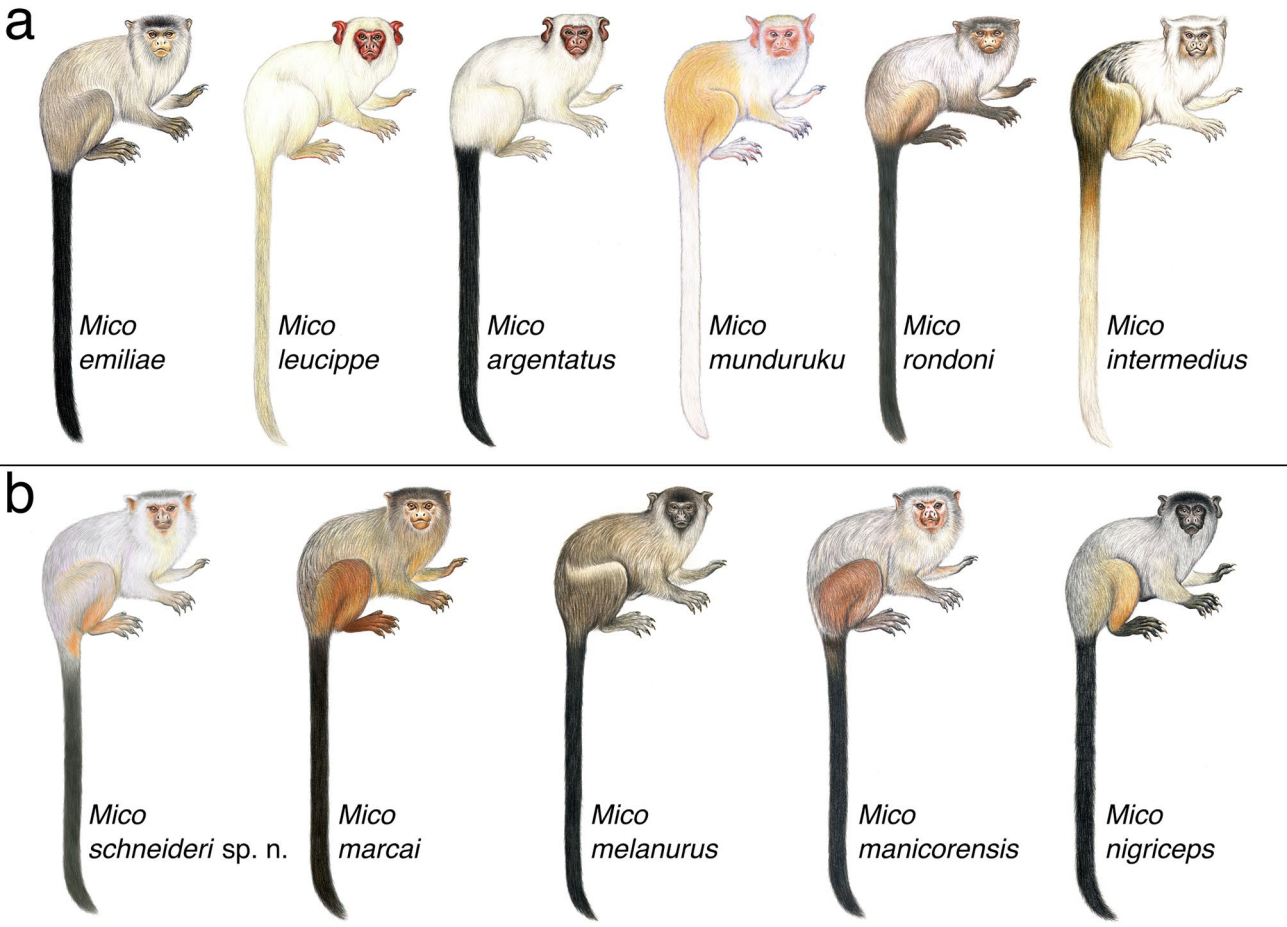
Two of the four lineages retrieved in genus *Mico*, based on morphological synapomorphies (data not shown) and phylogenomic analyses. **(a)** *Mico emiliae* lineage; **(b)** *Mico schneideri* sp. n. lineage. Illustrations: Stephen Nash.

We obtained a molecular dataset consisting of 2081 loci spanning 717,129 base pairs, representing an average sampling effort at the DNA level of 22,410 base pairs per specimen (n = 32). After analyses in PartitionFinder2, the 2081 loci were clustered into 421 partitions. In both Bayesian inference and Maximum Likelihood phylogenetic analyses, species monophyly and species-level relationships were highly supported (pp ≥ 0.99; bp > 70%) with exception of *M. emiliae*, *M. leucippe*, and *M. argentatus* (Fig. 4; Supplementary Fig. S7)*.* As expected, we retrieved four lineages or species groups in the genus *Mico* and found lineage membership patterns coherent with our predictions based on morphological synapomorphies*.* The Juruena–Teles Pires marmosets are monophyletic, sister to *M. marcai*, and both form a strongly supported lineage with *M. melanurus. Mico emiliae* is monophyletic, but nested within a clade that also includes paraphyletic *M. leucippe* and *M. argentatus.* These three species, together with *M. intermedius*, *M. munduruku*, and *M. rondoni* comprise the second major lineage of *Mico* (Fig. 5). A third lineage is comprised of *M. humeralifer* and *M. mauesi*, and *Mico saterei* was retrieved as an additional monotypic lineage. The clade formed by the Juruena–Teles Pires marmosets does not include *M. emiliae* specimens, nor is it sister to the *M. emiliae* clade; actually both taxa belong to separate lineages of *Mico.*

Our path sampling analysis clearly supports *M. argentatus*, *M. emiliae*, and *M. leucippe* as three separate species, considering that a Bayes factor > 10 is decisive^44^. Our results reject the hypothesis of only one species––*M. emiliae-M. leucippe*-*M. argentatus* (BF = 367.46)––and the hypotheses of two species––*M. argentatus* and *M. emiliae-M. leucippe* (BF = 145.08), *M. emiliae* and *M. leucippe-M. argentatus* (BF = 99.44), or *M. leucippe* and *M. argentatus-M. emiliae* (BF = 348.29). These results support the unambiguous diagnosis of these three species according to our morphological data.

### Distribution

**Figure 6:**
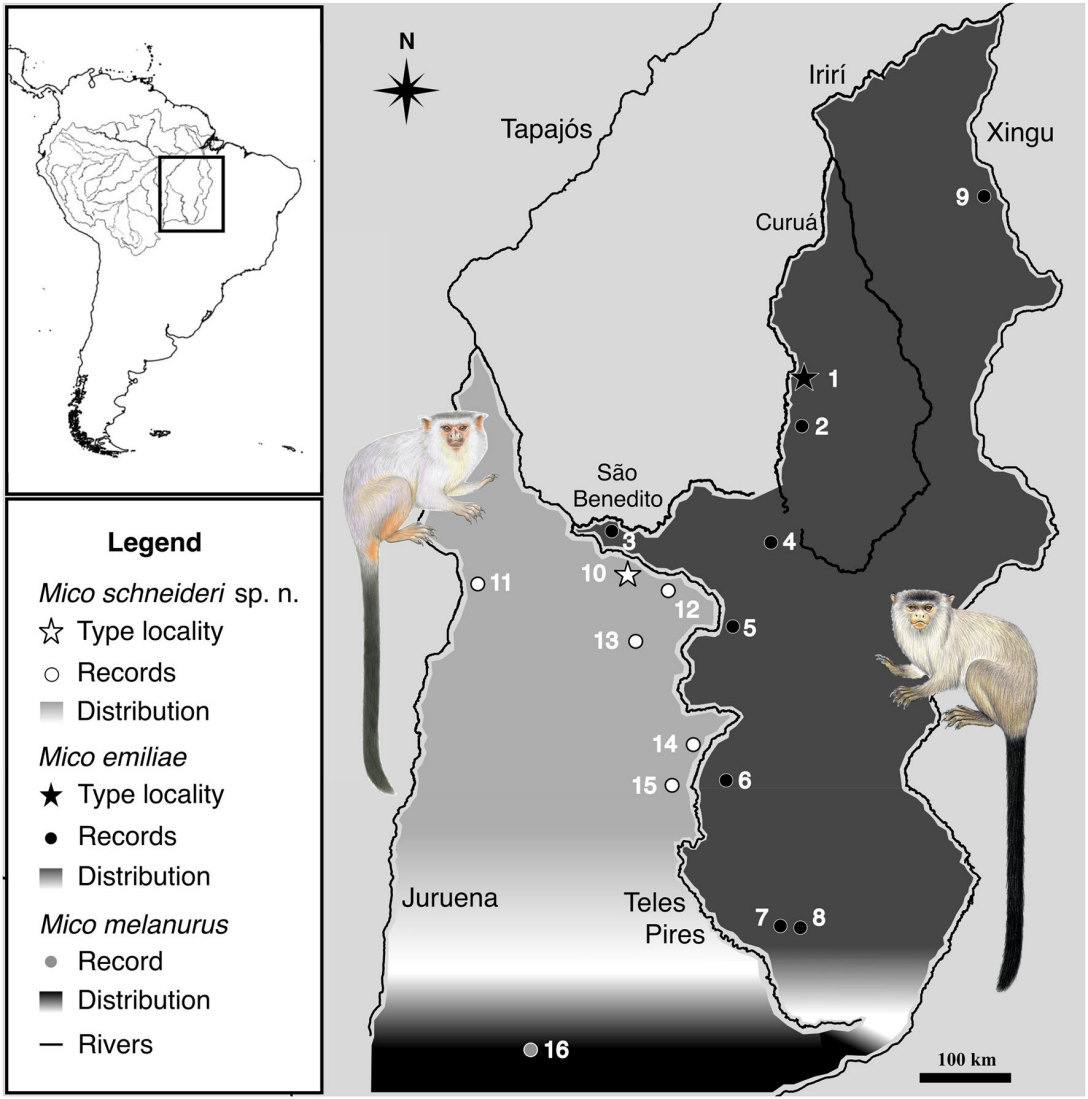
Geographic distribution of *Mico schneideri* sp. n. and *M. emiliae* (see Supplementary Table S8 for locality details). Illustrations: Stephen Nash.

The ranges of *M. emiliae* and Juruena–Teles Pires marmosets are separated by the Teles Pires River and in the headwaters of the Teles Pires River both are substituted by *M. melanurus* (Fig. 6; Supplementary Table S8)*.* There is no evidence of range overlap between the Juruena–Teles Pires marmosets and any other *Mico* species. The distributions of *M. emiliae* and Juruena–Teles Pires marmosets are allopatric; *M. emiliae* is parapatric with *M. leucippe* around the Cachimbo highlands^3^ and both *M. emiliae* and the Juruena–Teles Pires marmosets are parapatric with *M. melanurus* at the extreme south of their distributions. *Mico marcai*^46^, *M. rondoni*^21^ and *M. argentatus*^25^ are allopatric to *M. emiliae* and the Juruena–Teles Pires marmosets, being separated by rivers and by the ranges of other *Mico* species.

### Integrative approach in taxonomic hypothesis-testing and decision-making

The marmosets from the Juruena–Teles Pires interfluve have unique states of pelage colour characters when compared to all nominal species of *Mico* species, thus are clearly diagnosable, form a fully supported clade in our phylogenomic trees and are found only in the Juruena–Teles Pires interfluve, without evidence of range overlap with any other congeneric species. The criteria adopted here reject the null hypothesis that marmosets from the Juruena–Teles Pires interfluve are *M. emiliae* or another known species of *Mico*. We therefore describe them as a new species.

O﻿rder Primates Linnaeus, 1758

Family Callitrichidae Gray, 1821

Genus *Mico* Lesson, 1840

***Mico schneideri* sp. n.** Costa-Araújo, Silva-Jr., Boubli, Rossi, Hrbek & Farias

urn:lsid:zoobank.org:act:9B4FFB49-FC65-45CC-862B-AA97E1C8F5BA

**Holotype.** INPA 7293, tissue CTGA 5934, field number RCA 60, adult female, stuffed skin, skull, skeleton. This specimen was collected on April 2^nd^, 2016 in an urban forest fragment located in Paranaíta city (09°41′21″ S, 56°29′10″ W), on the left margin of Teles Pires River, Mato Grosso State, Brazil, by Rodrigo Costa Araújo.

**Type locality.** Paranaíta municipality, left margin of the Teles Pires River, northern Mato Grosso State, Brazil (09°41′21''S, 56°29′10''W)*.*

**Table 1:** Pelage colour characters from chromogenetic fields and their states in *Mico schneideri* sp. n. and in the morphologically and phylogenetically close related species.

	*Mico schneideri* sp. n	*Mico emiliae*	*Mico marcai*	*Mico rondoni*	*Mico argentatus*	*Mico melanurus*
Crown	Black	Black	Black	Black	White	Black
Head	White	Black and white	Light grey	Dark grey	White	Blackish brown
Mantle	Grey	Light brownish grey	Blackish grey	Dark grey	Silvery	Greyish brown
Forearms	Greyish cream	Blackish grey	Blackish light orange	Blackish dark brown	Silvery	Black
Hands	Blackish golden	Black	Greyish black	Black	Dark grey	Black
Saddle	Uniform lead	Light greyish brown	Blackish agouti	Pale brownish grey	Silvery	Blackish brown
Rump	Uniform lead	Light greyish brown	Blackish agouti	Pale brownish grey	Silvery	Blackish brown with two cream stripes
Underparts	Light greyish cream and orange	Brown agouti	Blackish ochre	Orangish black	Silvery	Orangish brown
Feet	Goldenish orange	Black	Blackish ochre	Orangish black	Dark grey	Black
Tail	Black	Black	Black	Black	Black	Black

**Paratypes.** Urban forest fragment, Paranaíta city, left margin of Teles Pires River (09°41′21″ S, 56°29′10″ W): INPA 7294, tissue CTGA 5935, field number RCA 61, adult female; INPA 7295, tissue CTGA 5936, field number RCA 62, adult male; both preserved in fluid and collected on April 2^nd^, 2016 by Rodrigo Costa Araújo. Urban forest fragment, Alta Floresta city, left margin of Teles Pires River (09°51′50′′ S, 56º04′20′′ W): MPEG 24595, field number RA 41, subadult male, stuffed skin, skull; MPEG 24596, field number RA 42, subadult male, stuffed skin, skull; both collected on October 16, 1995 by R. Alperin, R. Rodrigues, and N. Silva. Urban forest fragment, Alta Floresta city, left margin of Teles Pires River (09°53′04′′ S, 56°04′21′′ W): UFMT 3851, field number RVR 40, adult male, stuffed skin, skeleton, and tissue, collected on May 6, 2014 by Rogério Rossi. Peri-urban forest fragment, Alta Floresta, left margin of Teles Pires River (09°58′57′′ S, 56°04′21′′ W): UFMT 4833, field number RVR 43, adult male, stuffed skin, skeleton, and tissue; UFMT 3852, field number RVR 44, adult male, stuffed skin, skeleton, and tissue; UFMT 4834, field number RVR 45, adult male, stuffed skin, skeleton, and tissue, collected on May 6, 2014 by Rogério Rossi. Ourolândia, left margin of Teles Pires River (10°23′26″ S, 56°24′28′′ W): MPEG 24606, field number RA 63, male, stuffed skin, collected on October 22 1995; MPEG 24608, field number RA 68, adult male, stuffed skin, skull; MPEG 24609, field number RA 69, adult female, stuffed skin, skull; MPEG 24610, field number RA 70, adult female, stuffed skin, skull; MPEG 24611, field number RA 71, adult male, stuffed skin, skull; all collected on October 23 1995 by R. Alperin, R. Rodrigues, and N. Silva.

**Diagnosis.** Uniform lead colour on saddle and rump, and underparts cream-silvery with orange hues.

**Etymology.** The new species is named in honour of Professor Horacio Schneider, a pioneer, and a major contributor to the phylogenetic studies of Neotropical Primates, who humbly accepted to have this species named in his honour.

**Description of the holotype.** Hairs on the face are short, mostly white but also black or bi-banded black-white, distributed all over the face, denser in circumbucal area, rhinarium and sides of the face, increasing in size towards the sides of the face; eumelanic vibrissae on the rhinarium, supraorbital region and along the zygomatic bone. Face tegument eumelanic in the centre of the supraorbital area, around the eyes, along the sides and the middle of the nose thrill, on the rhinarium and circumbucal area; dark brown eyes. White and long hairs on outer and inner pinnae, longer and denser on the inner surface, partially covering the pinnae; ear tufts absent; eumelanic tegument on a large proportion of each ear, but paler than the eumelanic tegument of the face. White hairs surrounding the face and on the sides of the head, longer than on the face; white hair on the head, covering the lower portion of the pinnae; black crown, separated from facial hairs by a horizontal line of white hairs. Gray mantle. Dorsal forearms greyish cream, blackish golden hairs on hands; ventral forelimb hairs cream, white on ventral neck and chest. Cream hairs on the belly. Saddle and rump of a uniform lead colour. Hairs on underparts cream-silvery on the anterior region, grading to light orange towards the posterior area on the dorsal and ventral hind limbs; ventral hind limb hairs pure orange on the posterior area, whereas pure cream on the anterior area. Goldenish orange hairs on feet. Blacktail with orange hairs on the ventral surface of tail insertion, an inch in length. The tegument is slightly eumelanic on the ventral surface of hands, unpigmented on the ventral surface of feet; claw-like nails, curved dorsoventrally in all digits except the hallux, which bears a flat nail.

**Intra-specific morphological variation.** There is only minor individual variation among the marmosets from the Juruena–Teles Pires interfluve manifested as the conspicuousness of the orangish tone over the basic cream colour of hairs in the chest, belly, and ventral underparts. In prepared skins, such variation is little perceptible, as well as the orangish colour of the underparts, whereas in fresh specimens the ventral region of fore and hind limbs and belly hairs can show a bright and vivid orange-cream colour. A small amount of cream hair can be observed on and around the ears, chest, and in an even smaller quantity on the mantle in the paratypes INPA 7294 and INPA 7295. There is also a tonal variation in the colour of the hairs on hands, feet, and lower portion of underparts of the paratypes deposited at MPEG, attributable to fading in storage, varying from goldenish orange to light orange on feet and posterior area of underparts, and varying from golden to light yellow on hands (see Fig. 2, Supplementary Fig. S9).

**Geographic distribution.** *Mico schneideri* sp. n. is endemic to the Juruena–Teles Pires interfluve, southern Amazonia, Mato Grosso State, Brazil. The species distribution is limited by the Juruena River to the west and by the Teles Pires River to the east, proceeding north to their confluence. The southern portion of the species range is less well-defined, but it extends to the headwaters of the Juruena and Teles Pires rivers, but no further south than the city of Lucas do Rio Verde. In this region, the Amazonia biome transitions to the Cerrado biome, and thus parapatry is expected between *M. schneideri* sp. n. and *M. melanurus*, the only species of *Mico* known to occur in the Cerrado.

**Habitat.** Primary and secondary *terra firme* forests, and Amazonia-Cerrado transitional forests.

**Suggested vernacular names.** Schneider’s marmoset (English); sagui-de-Schneider (Portuguese).

*Mico emiliae* is morphologically diagnosable, monophyletic, and allopatric along most of its range. *Mico argentatus* and *M. leucippe* are morphologically diagnosable and, although they were not retrieved as monophyletic in our phylogenetic inferences, path sampling analysis of genomic data associated with morphology and distribution data provide decisive support for their recognition as distinct species. *Mico argentatus* is allopatric on the east bank of the Xingu River and on the west bank of the Xingu River, this species is parapatric to *M. leucippe*––which is, in turn, apparently restricted to a narrow area between the Jamanxim and the Irirí-Curuá Rivers^3,25^. The distribution of *M. leucippe* is not limited by any conspicuous geographical barrier that could prevent introgression with *M. argentatus* or *M. emiliae*.

## Discussion

In the early twentieth century, the German ornithologist Emilie Snethlage conducted two field expeditions across the forests of the Tapajós–Xingu interfluve, southern Amazonia, Pará State, Brazil. In the second expedition, Snethlage discovered ‘small and enchanting black-headed marmosets, closely related to [*M. argentatus*], and certainly belonging to a new species’^47^. She collected two of these marmosets at “Maloca, upper Curuá River”, which became the type specimens and the type locality of *M. emiliae*^12^^‚^. Since then, *M. emiliae* accumulates a history of taxonomic uncertainties, which permeates the taxonomy of *M. argentatus, M. marcai, M. rondoni*, and﻿ *Mico schneideri *sp. n., and thus the pattern of species diversity in this genus. None of these five marmoset species had been subjected to robust taxonomic assessments to date due to a scarcity of specimens for morphology-based studies and the lack of tissue samples for molecular phylogenetic analysis*.*

The two marmosets Snethlage collected at Maloca^12^ and the three specimens collected at the Cachimbo highlands^48^ were, until now, the only specimens known from the region of the type locality of *M. emiliae* in the Tapajós–Xingu interfluve. Following its original description^12^, *M. emiliae* was considered as invalid or as subspecies of *M. argentatus*^13–18^. Decades later, when these two species were considered valid, morphologically divergent marmosets from three other interfluvial regions in Amazonia were identified as *M. emiliae*^19^ leading to questions about the taxonomic identity of these populations and of *M. emiliae*. Moreover, the type locality of *M. emiliae* is uninformative: *maloca* is the name of the traditional dwellings of the Chipaya and Curuahy, the native peoples from the Curuá River basin that Snethlage travelled with––and there are several *malocas* depicted on her expedition map^47^. Until now, no geographical coordinates had been proposed for the type locality of *M. emiliae* because no field research was conducted on this species subsequent to Snethlage’s expedition.

To resolve these uncertainties, we first focused on finding *M. emiliae* type locality. Initially, we studied Snethlage’s narrative, the map of her field expedition, and the labels of *M. emiliae* type specimens, which she handwrote. Based on these sources we identified only one “Maloca”––which matches exactly the spelling on the map and the labels of type specimens. Based on this deduction and using local relief references we restricted the type locality of *M. emiliae* and extracted the geographic coordinates of this specific *maloca* which is located not far south of the mouth of the Curuaés stream on the east bank of the Curuá River within the Tapajós–Xingu interfluve (09°41′21′′ S, 56°29′10′′ W).

We then conducted field expeditions to the type locality of *M. emiliae* as here defined, where we recorded groups and collected a specimen that had the same morphology and pelage coloration as the type specimens of *M. emiliae*, confirming the location of this specific *maloca* where Snethlage was in 1911. We also conducted several field expeditions within the Tapajós–Xingu to collect additional distribution records, specimens, and tissue samples of *M. emiliae*, as well as within all other main interfluves across southern Amazonia to obtain these materials and data from all *Mico* species. The surveys included the localities where specimens of *Mico schneideri* sp. n. were previously collected and the type localities of *M. marcai* and *M. rondoni*, so our datasets included topotypic material from these four species.

Combining data collected from specimens obtained in the field and examined in museum collections, we were able to demonstrate that *M. emiliae* is an evolutionarily independent lineage composed of specimens distinctive in pelage colour, which occur in an area largely restricted by the Rivers Curuá, Irirí, São Benedito and Teles Pires in the west and north, and by the Xingu River at the east. We also identified the type locality of *M. emiliae* for which we provided exact geographical coordinates, and redefined its distribution––extending it 200 km south. The southern limit of the distribution remains uncertain, but it probably extends to the northern edge of the Cerrado biome––where in further surveys we expect to find a small contact zone between *M. emiliae* and *M. melanurus* at the headwaters of the Teles Pires and Xingu rivers. We deem it probable that *M. emiliae* also occurs in the Cerrado savanna vegetation, as we found groups of this species in Amazonian white-sand savanna ecosystems––scrubland and *campinarana*^49^ vegetation*––*in our surveys of the Amazonian forests of the Tapajós–Xingu interfluve.

The marmosets from the Juruena–Teles Pires interfluve herein described as *Mico schneideri* sp. n. are, in fact, sister to *M. marcai* and not to *M. argentatus*^50^ nor nested within *M. emiliae*^22^*. Mico schneideri* sp. n. and *M. marcai* are diagnosable by pelage colour patterns, are reciprocally monophyletic, belong to a different lineage than *M. emiliae*, and the distributions of both species are allopatric. Therefore, *Mico schneideri* sp. n. is a valid species. Further field surveys are necessary to better define the southern limit of the geographical distribution of this new species. As there is no evidence that *Mico schneideri* sp. n. occurs in Amazonian white-sand savanna vegetation, we do not expect to find it in the Cerrado biome.

The inclusion of topotypic specimens of *M. emiliae* and *M. rondoni* in a molecular phylogenetic analysis for the first time also confirmed the hypothesis that *M. rondoni* is a distinct and valid species. Although historically confounded with *M. emiliae*^19,20^, *M. rondoni* is monophyletic, has a distribution restricted to the Guaporé–Ji Paraná interfluve which is allopatric to *M. emiliae*, and is characterized by a distinctive pelage colour pattern when compared to other congenerics. *Mico rondoni* was described based on pelage colour and a molecular phylogeny^21,50^ but, actually, the specimens considered as *M. emiliae* in the phylogeny belong to *Mico schneideri* sp. n.

*Mico argentatus* is also clearly diagnosable by pelage colour patterns. Although *M. argentatus* and *M. leucippe* were retrieved as paraphyletic in this and in a previous study^3^, our path sampling analysis provides decisive evidence for their recognition as separate evolutionary lineages. The path sampling analysis collects the marginal likelihoods of phylogenetic trees differing only in the number of species, thus isolating the effect of number of species in the likelihoods; the marginal likelihoods are then compared using the Bayes factor to test differences in the number of species assumed a priory. *Mico argentatus* is allopatric to other congeneric species on the east bank of Xingu River and, on the west bank of the Xingu River, *M. argentatus* is separated from *M. emiliae* by the Irirí River, and by the range of *M. leucippe*^3,25^. Nonetheless, *M. argentatus* is parapatric to *M. leucippe* on the west bank of Xingu River and without a clear geographical barrier that could prevent gene flow between populations of both taxa. Moreover, there is no barrier also between populations of *M. leucippe* and *M. emiliae* around the Cachimbo highlands.

We consider that our morphological and phylogenomic results provide strong evidence to recognize *M. argentatus* and *M. leucippe* as distinct species. Given that most closely related Amazonian primate taxa are separated by physical barriers, we are now exploring the biological and evolutionary processes underlying the existence of such distinct but parapatric marmoset species in a region where there are no apparent physical barriers to gene flow. The taxonomy and distribution of *M. marcai* is also not well-resolved. It is unclear if this species and *M. manicorensis* are distinct from each other and from *M. nigriceps*. All three taxa are morphologically similar and occur in geographically adjacent areas within the Ji-Paraná–Aripuanã interfluve^25^.

The centenary uncertainties in the taxonomy of *M. emiliae* emerged from a case of pseudo-cryptic diversity in Neotropical primates. Pseudo-cryptic species are morphologically distinctive^51,52^ but such distinctness is overlooked due to methodological inadequacies^53^. Previous taxonomic studies on this species relied on pelage colour patterns obtained from few individuals, some of faded pelage colour, as the sole source of information on which taxonomic decisions were based. The low number of specimens and the few known distribution records in previous studies probably caused the taxonomic confusion surrounding *M. emiliae*, *M. argentatus*, *M. marcai*, *M. rondoni*, and *Mico schneideri* sp. n., which in reality are unambiguously diagnosable and distinctive in pelage colour as shown here. *Mico schneideri* sp. n. was hidden at plain sight: the eight specimens from the Juruena–Teles Pires interfluve, stored in the Museu Paraense Emílio Goeldi since 1995, have been consistently misidentified as *M. emiliae*^21,22,50^ and are here designated as *Mico schneideri* sp. n. paratypes.

Using an integrative taxonomic analysis, carried out with data obtained from a greater number of specimens than in any previous study, we were able to accurately diagnose *Mico schneideri* sp. n., *M. argentatus*, *M. emiliae*, *M. marcai*, and *M. rondoni*. The new tissue samples and expanded occurrence data were paramount in allowing a first phylogenomic inference of evolutionary relationships and a robust taxonomic assessment of all these species, as well as a refined understanding of the distribution of *M. emiliae* and *Mico schneideri* sp. n. Our research highlights the importance of fieldwork, scientific collections, and the use of multiple data sources within an integrative taxonomic framework of delimiting primate species^3,4^. Fieldwork is paramount to obtain specimens, samples, and field records, to overcome the scarcity of such materials and data currently available in museums and literature, and to allow the accurate distinction between species and population patterns in morphology and molecular data, as well as to define species geographic distributions.

## Conclusion

A century of taxonomic uncertainties surrounding *M. emiliae* has hopefully come to an end with the description of *Mico schneideri* sp. n. from the Juruena–Teles Pires interfluve and the recognition of *M. argentatus*, *M. emiliae*, *M. leucippe*, *M. marcai*, and *M. rondoni* as distinct species. Nonetheless, the existence of *M. argentatus* and *M. leucippe* as distinct species in the absence of physical barriers to gene flow on the west bank of Xingu River is intriguing and the biological processes underlying such scenario deserve further clarification. More studies are also needed on the taxonomy and distribution of *M. marcai*, *M. manicorensis*, and *M. nigriceps* to clarify the species diversity in genus *Mico.* Criteria-driven test of species hypotheses based on data from pelage colour, genomic DNA, and geographic distribution can resolve the taxonomy and evolutionary relationships of Neotropical primates while reducing subjectivity and adding rigour to the decision-making process. Fieldwork is a critical component of such studies in providing information on phenotypic variation, distribution records, samples and specimens not foreseen in earlier collections and is necessary to further advance the knowledge of diversity and distribution of Neotropical primates. Our research indicates that there are 16 *Mico* species, 19 marmoset species in Amazonia (including the dwarf marmoset *Callibella humilis* and two pygmy marmoset species *Cebuella pygmaea* and *C. niveiventris*), and 25 marmoset species in Neotropics (including six *Callithrix* species from eastern Brazil). Delimiting taxa and understanding their distributions is fundamental to support the implementation of effective conservation strategies for Amazonian primates. Such efforts are especially urgent in the Amazonian arc of deforestation, where 52 primate species are largely overlooked by the scientific and conservation community but face high rates of habitat loss^54^. Primates are effective conservation programme flagship species^55^, play an important role in ecosystem functioning^56^ and are the most threatened group of vertebrates in the world^2^. Therefore, characterizing primate species diversity and distribution in the Amazonian arc of deforestation is a necessary first step on which an entire science-based conservation effort depends and which lends support to biodiversity conservation in this region before the entire biome reaches an environmental point of no return^57^.

## Supplementary Information

Supplementary Information.

## Data Availability

Genomic data generated for this study have been deposited in Genbank (PRJNA552061) and alignment and other data generated during and/or analysed during the current study are available at https://github.com/legalLab/publications.

## Code availability

This published work and the nomenclatural acts it contains have been registered in ZooBank, the online registration system for the International Code of Zoological Nomenclature, under the ZooBank Life Science Identifier (LSID) urn:lsid:zoobank.org:pub:B43E02D9-F8CD-4ADA-9B86-CD9378C51723 and urn:lsid:zoobank.org:act:9B4FFB49-FC65-45CC-862B-AA97E1C8F5BA, respectively, and can be resolved and the associated information viewed through any standard web browser by appending the LSID to the prefix ‘http://zoobank.org/’.
