# Supplementary material for: An integrative analysis uncovers a new, pseudo-cryptic species of Amazonian marmoset (Primates: Callitrichidae: Mico) from the arc of deforestation
Source: Sci Rep. 2021 Aug 2;11:15665. doi: 10.1038/s41598-021-93943-w (PMC8328995; doi:10.1038/s41598-021-93943-w)
Supplement: Supplementary file 1 — Supplementary Information. [file 41598_2021_93943_MOESM1_ESM.pdf]

An integrative analysis uncovers a new, pseudo-cryptic species of Amazonian marmoset (Primates: Callitrichidae: *Mico*) from the arc of deforestation

Rodrigo Costa-Araújo, José Silva-Jr., Jean Boubli, Rogério Rossi, Gustavo Canale, Fabiano Melo, Fabrício Bertuol, Felipe Silva, Diego Silva, Stephen Nash, Iracilda Sampaio, Izeni Farias, and Tomas Hrbek

Supplementary Table S1. Primate species currently known to occur in southern Amazonia, the arc of deforestation, and their conservation status according to IUCN[10].

Species	Conservation Status
Alouatta ululata	**Endangered**
Alouatta belzebul	**Vulnerable**
Alouatta discolor	**Vulnerable**
Alouatta caraya	Near Threatened
Alouatta nigerrima	Least Concern
Alouatta sara	Least Concern
Alouatta seniculus	Least Concern
Aotus infulatus	Least Concern
Aotus nigriceps	Least Concern
Ateles chamek	**Endangered**
Ateles marginatus	**Endangered**
Callibella humilis	Least Concern
Cebus kaapori	**Critically Endangered**
Cebus unicolor	**Vulnerable**
Chiropotes satanas	**Endangered**
Chiropotes albinasus	**Vulnerable**
Chiropotes utahickae	**Vulnerable**
Lagothrix cana	**Endangered**
Leontocebus weddelli	Least Concern
Mico leucippe	**Vulnerable**
Mico marcai	**Vulnerable**
Mico munduruku	**Vulnerable**
Mico rondoni	**Vulnerable**
Mico humeralifer	Near Threatened
Mico melanurus	Near Threatened
Mico nigriceps	Near Threatened
Mico acariensis	Least Concern
Mico argentatus	Least Concern
Mico chrysoleucos	Least Concern
Mico emiliae	Least Concern

Species	Conservation Status
Mico intermedius	Least Concern
Mico mauesi	Least Concern
Mico saterei	Least Concern
Pithecia mittermeieri	**Vulnerable**
Pithecia rylandsyi	**Vulnerable**
Plecturocebus grovesi	**Critically Endangered**
Plecturocebus vieirai	**Critically Endangered**
Plecturocebus brunneus	**Vulnerable**
Plecturocebus parecis	Near Threatened
Plecturocebus baptista	Least Concern
Plecturocebus bernhardi	Least Concern
Plecturocebus cinerascens	Least Concern
Plecturocebus donacophilus	Least Concern
Plecturocebus hoffmannsi	Least Concern
Plecturocebus moloch	Least Concern
Saguinus ursulus	**Vulnerable**
Saguinus niger	**Vulnerable**
Saimiri ustus	Near Threatened
Saimiri boliviensis	Least Concern
Saimiri collinsi	Least Concern
Sapajus apella	Least Concern
Sapajus cay	Least Concern

Supplementary Table S2. Geographical coordinates (*datum* WGS 84) here attributed for the localities of *Mico emiliae* specimens and for specimens historically identified as *M. emiliae* but belonging to distinct taxa (see Figure 1).

Localities	Coordinates	Taxa
Maloca, upper Curuá River[12]	07°36'59"S, 54°52'00"W	*Mico emiliae*
Peixoto de Azevedo River[17]	10°13'02"S, 55°21'41"W	
Serra do Cachimbo[48]	09°22'00"S, 55°00'00"W	
Roosevelt River[19,20]	07°33'50"S, 60°41'12"W	*Mico marcai*
Nova Brasília[19 †]	10°53'15"S, 61°54'35"W	*Mico* aff. *marcai*
Urupá River[19 †]	10°53'10"S, 61°57'15"W	*Mico* aff. *melanurus*
Samuel dam[20,21]	08°45'00"S, 63°28'00"W	*Mico rondoni*
Santa Bárbara[20,21]	08°46'35"S, 63°53'44"W	
Alta Floresta[21 †]	09°51'50"S, 56°04'20"W	*Mico schneideri* sp. n.
Ourolândia[21 †]	10°23'26"S, 56°24'28"W	

[†] Taxa identification revised from the original source.

Supplementary Table S3. Framework adopted in this study for taxonomic assessment and decision-making[28] and following the monophyly and diagnosability conceptualization of the phylogenetic species concept[30].

Sources of information	Criteria	Single source	Integrated sources
Morphology data<br>Pelage pigmentation	Diagnosability	H0 / H1	
Molecular data<br>Nuclear genome	Monophyly	H0 / H1	H0 / H1
Distribution data<br>Localities of occurrence	Allopatry<br>Parapatry	H0 / H1	

Supplementary Figure S4. Schematic representation of the chromogenetic fields[32] here examined as morphological characters[3]. Illustration: R. Costa-Araújo.

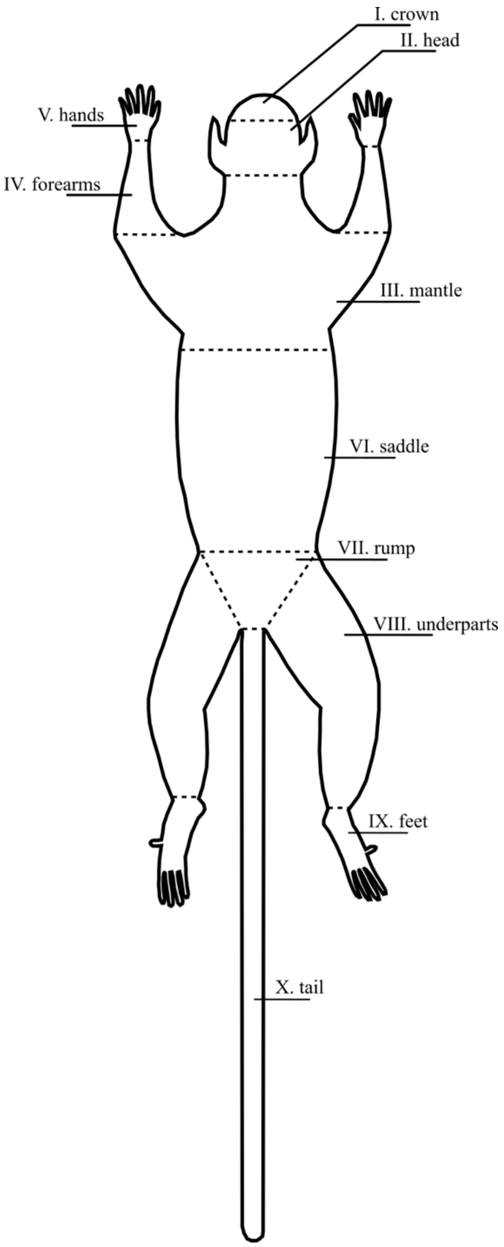

Supplementary Table S5. Prepared skins and specimens in fluid examined in the mammal collections of the Field Museum of Natural History (FMNH), Instituto Nacional de Pesquisas da Amazônia (INPA), Museu Paraense Emilio Goeldi (MPEG), Museu Nacional. Universidade Federal do Rio de Janeiro (MN), Museu de Zoologia da Universidade de São Paulo (MZUSP), Natural History Museum London (NHM), Naturstorisches Museum Wien (NMW), Universidade Federal de Rondônia (UNIR), and Universidade Federal do Mato Grosso (UFMT) for collection of morphological data. The type specimens are indicated with bold letters and the specimens obtained for this study are indicated with asterisk.

Taxon	Specimens
Mico acariensis (n=6)	INPA: **3931**; MPEG: 45578*, 45579*, 45580*, 45581*, 45582*.
Mico argentatus (n=171)	FMNH: 19499, 19533, 50839, 50840, 50841, 92177, 92178, 92179, 92180; INPA: 2473; MPEG: 29, 38, 151, 154, 156, 157, 162, 163, 164, 165, 165, 166, 269, 328, 336, 6878, 8951, 8952, 8982, 8983, 8984, 8985, 8986, 8987, 9200, 9201, 9202, 9203, 9204, 9205, 9206, 9207, 9208, 9212, 10021, 10022, 10023, 10024, 10025, 10026, 21373, 21374, 21375, 21376, 21377, 21378, 21379, 21380, 21381, 21382, 21383, 21384, 21385, 21386, 21387, 21388, 21389, 21390, 21391, 21392, 21393, 21394, 21414, 21630, 21631, 21632, 21633, 21634, 21635, 21636, 21637, 22922, 22923, 22924, 22925, 22926, 22927, 22928, 22929, 23156, 23157, 23158, 45605*, 45606*, 45607*, 45608*, 45609*; MN: 2412, 2834, 2845, 2846, 2847, 2878, 4508, 5116, 5718, 5946, 5953, 5954, 11571, 11572, 11573, 11574, 11575, 11576, 11577, 11915, 23826; MZUSP: 3588, 3589, 3591, 3593, 3594, 3595, 3596, 4313, 4829, 4833, 4840, 4865, 4899, 4900, 4901, 4902, 4903, 4904, 4905, 4906, 4907, 4908, 4909, 4910, 4911, 4913, 4914, 4915, 4916, 4918, 4940, 4959, 4964, 4965, 4966, 4967, 4968, 4969, 4970, 4971, 4972, 4973, 4974, 4975, 5007, 5026, 6633, 11272, 11307, 11308, 11367, 11407, 11307, 11308.
Mico chrysoleucos (n=74)	FMNH: 50821, 50822, 50823, 50825, 50826, 50827, 50828, 50829, 50830, 50831, 50832, 50833, 50834; INPA: 4110, 4038, 4039, 7388*, 7389*, 7390*; MPEG: 237, 544, 23064, 45576*, 45577*, 45587*, 45590*, 45591*, 45610*; MN 2835, 2836, 2837, 5947, 5948, 5949, 5950, 5951, 5952, 6054, 6055, 6061, 6062, 6095; MZUSP: 4210, 4211, 4884, 4885, 4886, 4887, 4888, 4890, 4892, 4893, 4894, 4976, 5006, 5008, 5009, 5018, 5019, 5020, 5022, 5028, 5029, 5030, 11246, 11409, 11410, 11411, 13466, 13467; NMW: **ST 970**, **B 3455**, **B 3456**, **B 3457**.
Mico emiliae (n=11)	INPA: 7287*, 7288*, 7289*, 7290*; MPEG: **170**, 37807, 37808, 37809, 45566*; MZUSP: 35106; NHM: **20.7.14.12**.
Mico humeralifer (n=122)	FMNH: 19508, 92165, 92166, 92167, 92168, 92169, 92170, 92171, 92172, 92173; INPA: 4111, 4083, 7385*, 7386*, 7282*; MPEG: 30, 172, 173, 174, 175, 178, 265, 266, 267, 268, 337, 1384, 9213, 21397, 21398, 21399, 21400, 21401, 21402, 21403, 21404, 21405, 21406, 21407, 21408, 21409, 21410, 21411, 21412, 38387, 38504, 38505, 39474, 39475, 40984, 44282, 44283, 5904*, 5905*; MN 2838, 2839, 2840, 11000, 23818; MZUSP: 3577, 3578, 3579, 3580, 3582, 3584, 3585,

Taxon	Specimens
	3586, 4927, 10095, 10096, 10097, 10098, 10099, 11249, 11250, 11251, 11252, 11253, 11254, 11255, 11256, 11257, 11258, 11259, 11260, 11261, 11262, 11263, 11264, 11265, 11266, 11268, 11269, 11270, 11271, 11294, 11297, 11298, 11299, 11300, 11301, 11302, 11303, 11304, 11306, 11309, 11310, 11312, 11332, 11333, 11347, 11348, 11349, 11350, 11351, 11356, 11357, 11358, 11360, 11393, 11396, 11397, 11398, 11399, 11400, 11401, 11412, 11413, 18866.
Mico intermedius (n=10)	MPEG: **8156**, **12599**, 23065, 45585*, 45592*, 45594*, 45595*, 45598*, 45600*; MN 2850.
Mico leucippe (n=30)	FMNH: 92174, 92175, 92176; MPEG: 43661, 45563*, 45564*, 45565*, 45567*, 45568*, 45569*; MN 4798; MZUSP: 3598, 9964, 9965, 10093, 10094, 11248, 11279, 11280, 11281, 11291, 11295, 11296, 11305, 11311, 11361, 11394, 11402, 11403; NHM **9.3.9.2**.
Mico manicorensis (n=1)	INPA: **2511**.
Mico marcai (n=9)	MPEG: 45638, 45640, 42807, 42808, 43008, 45641; MN 2851, 2856, 2857.
Mico mauesi (n=11)	INPA: 4105, 4106, 4107, 4108, 4109, 4082; MPEG: **22177**, 23962, 23963, 23964; MZUSP: 29027.
Mico melanurus (n=54)	FMNH: 26730, 44859, 51888; INPA: 7296*, 7283*, 7383*; MPEG: 15266, 15267, 21395, 21396, 45571*; MN 2848, 2849, 2852, 2853, 2854, 2855, 2858, 2859, 5829, 5843, 5845, 5847, 5849, 23827, 24778, 24912, 24913, 25008; MZUSP: 3367, 3368, 3369, 3370, 3376, 4263, 4264, 4265, 4266, 6327, 6328, 6329, 6330, 6332, 6333, 24734; NMW: ST 1578, B 3447, B 3448, B 3449, B 3451, B 3758; UNIR: 192, 346, 356.
Mico nigriceps (n=17)	MPEG: **21996**, **21997**, **21998**, **21999**, 22955, 22956, 22957, 22958, 22959, 22960, 22961, 22962, 45614*, 45615*, 45616*, 45617*, 45618*.
Mico rondoni (n=46)	MPEG: 21365, **21366**, **21367**, **21646**, **21647**, 21648, 21649, 21650, 21651, 21652, 21653, 21654, 21655, 21656, 21657, 21658, **21659**, **21660**, 21885, **21886**, 21887, 21888, 21889, 21891, 21892, **21893**, **21894**, 21895, **21896**, **21897**, 21898, 28691, 45620*; MN 28486; MZUSP: 20142; UNIR: 15, 26, 48, 78, 159, 162, 165, 396, 556, 639, 158.
Mico saterei (n=14)	INPA: 4040, 4101, 4102, 4103, 4104, 4082, 5672; MPEG: **23955**, **23956**, **23957**, **23958**, **23959**, **23960**, **23961**.
Mico schneideri sp. n. (n=14)	INPA: **7293***, **7294***, **7295***; MPEG: **24595**, **24596**, **24606**, **24608**, **24609**, **24610**, **24611**; UFMT: **3851**, **3852**, **4833**, **4834.**

Supplementary Table S6. Specimens stored in the collections of the Coleção de Tecidos de Genética Animal (CTGA) of the Universidade Federal do Amazonas, Instituto Nacional de Pesquisas da Amazônia (INPA), Instituto de Desenvolvimento Sustentável Mamirauá (IDSM), Museu Paraense Emílio Goeldi (MPEG), Universidade Federal do Mato Grosso (UFMT), and Universidade Federal de Rondônia (UNIR) sampled to generate genomic DNA sequences (ddRAD) for phylogenetic analysis.

Taxon	Specimens
Callibella humilis	INPA 4090
Callithrix jacchus	CTGA 7017*
Cebuella niveiventris	CTGA 170
Mico argentatus	CTGA 434, 6015, 6016; MPEG 45605*†
Mico emiliae	INPA 7287*, 7289*, 7290*†; MPEG 45566*
Mico humeralifer	INPA 7282*
Mico intermedius	UFMT 4705; IDSM 792
Mico leucippe	CTGA 5912*; MPEG 45564*, 45575*†, 45568*
Mico marcai	MPEG 43008, 45638, 45645
Mico mauesi	CTGA 5881*
Mico melanurus	INPA 7283*, 7383*; UNIR 346
Mico munduruku	INPA 7284*, 7382*; MPEG 45559*, 45622†
Mico rondoni	MPEG 45620*; UNIR 556
Mico saterei	INPA 4101
Mico schneideri sp. n.	INPA 7293*, 7294*; MPEG 45573*; UFMT 4834.

*Specimens obtained for this research.
†Specimens included in path sampling analysis.

Supplementary Figure S7. Maximum Likelihood phylogeny of the genus *Mico* inferred with ddRAD data, indicating the four main lineages of this genus in distinct colours (black lines are outgroups). Clades supported are indicated by black circles, unresolved branches (<70% bootstrap proportion) are in white circles. Illustrations: Stephen Nash.

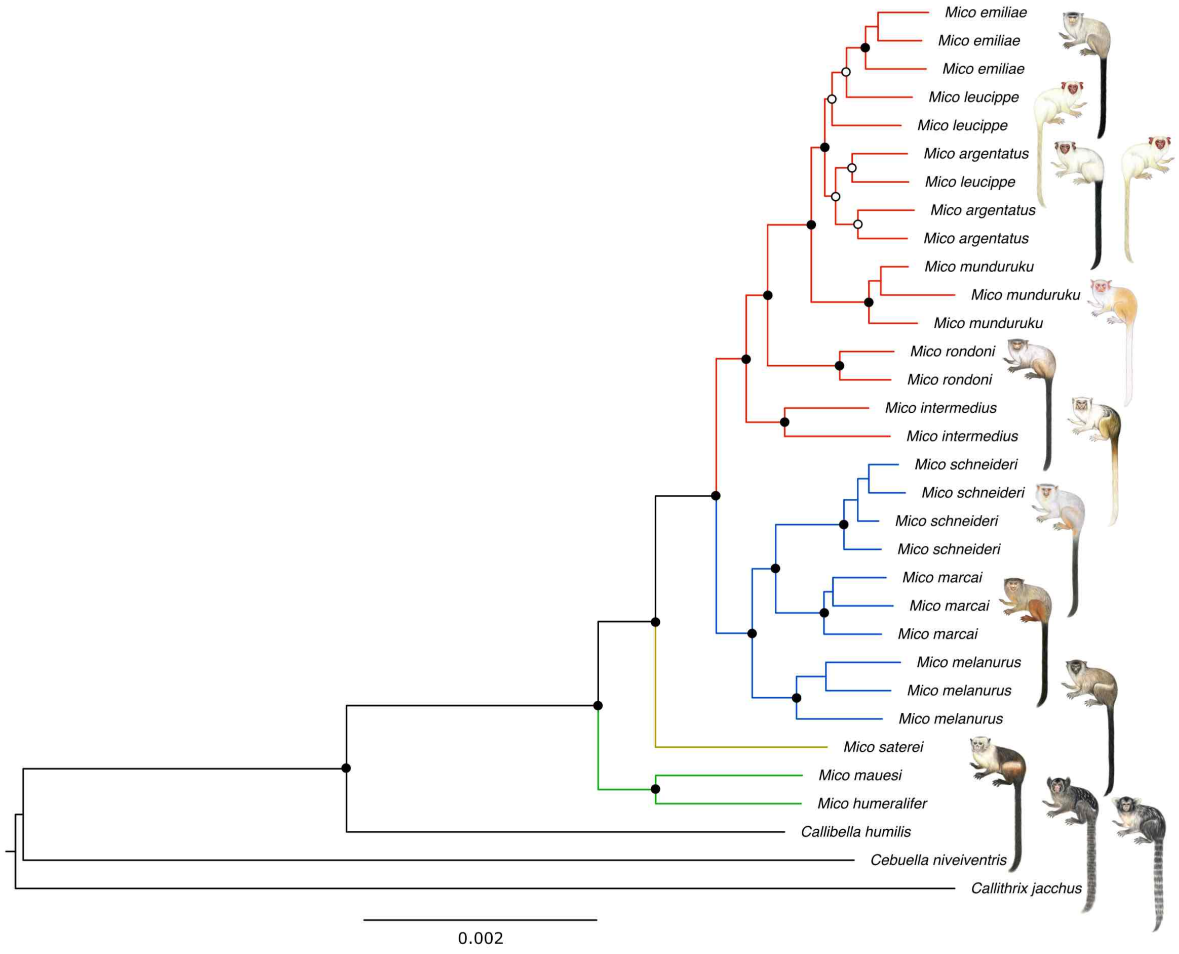

Supplementary Table S8. Geographical coordinates (*datum* WGS 84) here attributed to localities used to delimit species geographical distributions (see Figure 6). Bold=type specimens; asterisks=specimens examined for morphology data; crosses=specimens sampled for genomic data.

	Localities	Coordinates	Types of record	References	Specimen codes
Mico emiliae					
1	Maloca, upper Curuá River—type locality	07°36'59''S, 54°52'00''W	preserved specimens	12, this paper	**MPEG 170***; **NHM 20.7.14.12***
2	Upper Curuá River, right margin	08°07'32''S, 54°59'46''W	preserved specimens	this paper	MPEG 45566*†
3	Thaimaçu, left margin of São Benedito River	09°03'06''S, 56°35'12''W	preserved specimens	58, this paper	INPA 7288*, 7289*†, 7290†*
4	Cachimbo highlands	09°22'00''S, 55°00'00''W	preserved specimens	48	MPEG 37807*, 37808*, 37809*
5	Peixoto de Azevedo River	10°13'02''S, 55°21'41''W	survey	17	-
6	Sinop, right margin of Teles Pires River	11°50'10''S, 55°29'59''W	preserved specimen	this paper	INPA 7287*†
7	von der Steinen River	13°15'41''S, 54°52'02''W	survey	this paper	-
8	Ronuro River	13°18'19''S, 54°33'42''W	survey	this paper	-
9	Serra do Pardo National Park	05°48'03''S, 52°37'58''W	survey	59	-
Mico schneideri sp. n.					
10	Paranaíta city—type locality	09°41'21''S, 56°29'10''W	preserved specimen	this paper	**INPA 7293***†, **7294***†, **7295***
11	Juruena	09°47'41''S, 58°11'43''W	survey	this paper	-
12	Alta Floresta	09°58'57''S, 56°04'21''W	preserved specimen	this paper	**MPEG 24595***, **24596***, **45573***†; **UFMT 3851***, **3852***, **4833***, **4834***†
13	Ourolândia	10°23'26''S, 56°24'28''W	preserved specimen	this paper	**MPEG 24606***, **24608***, **24609***, **24610***, **24611***
14	Sinop, left margin of Teles Pires River	11°27'48''S, 55°33'48''W	survey	this paper	-
15	Left margin of Verde River	11°53'14''S, 55°52'18''W	survey	this paper	-
Mico melanurus					
16	Tangará da Serra	14°18'00''S, 57°44'00''W	survey	60	-

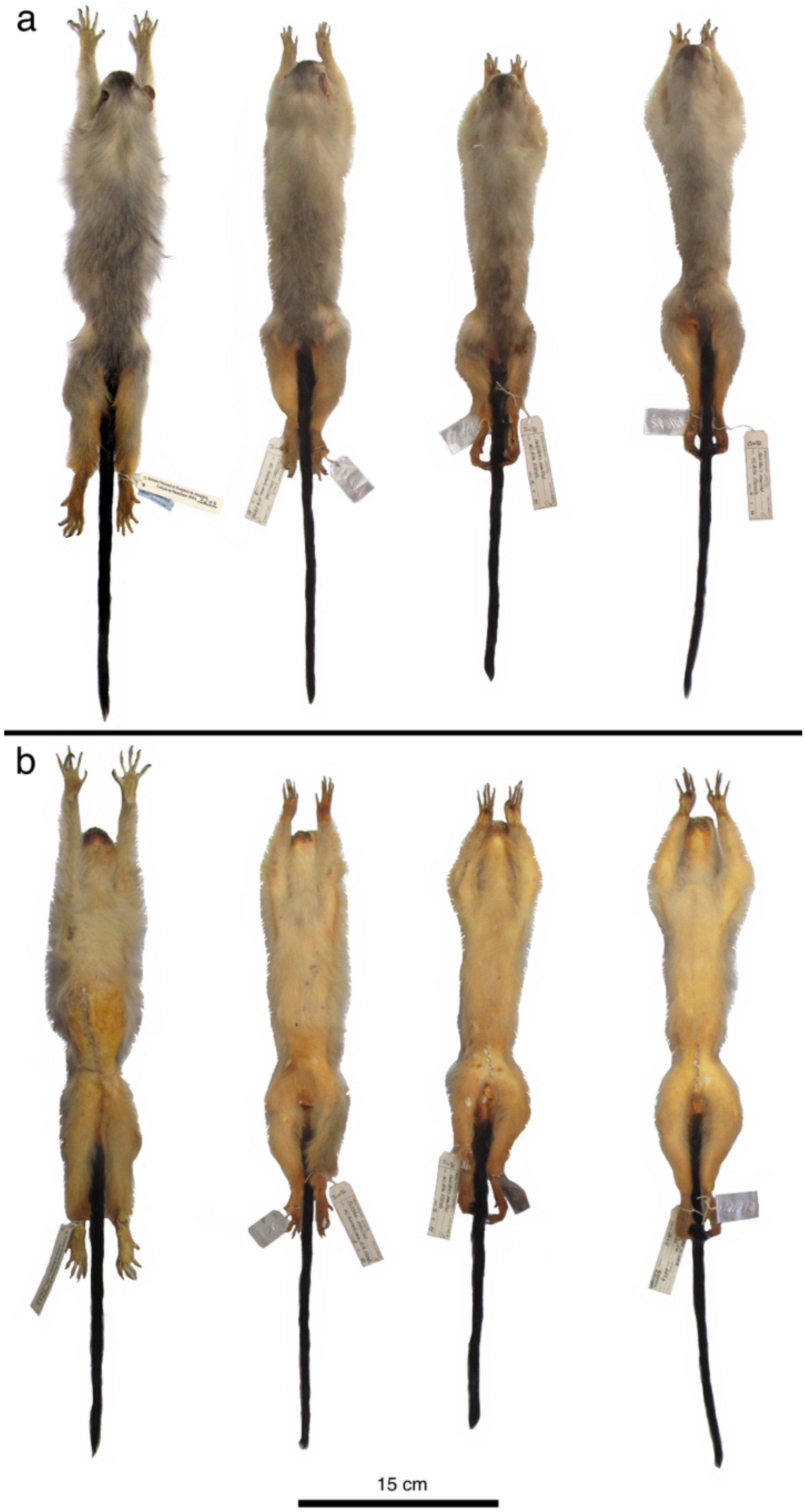

Supplementary Figure S9. Intraspecific variation in pelage colour of *Mico schneideri* sp. n.: a. dorsal view; b. ventral view. Left to right: holotype (INPA 7293) and paratypes MPEG 24608, 24595, 24596.
